# Supplementary material for: A Post-Operative Follow-Up of an Endangered Saltwater Fish Lensectomy for Cataract Management in a Public Aquarium: A Case Series
Source: Vet Sci. 2023 Oct 8;10(10):611. doi: 10.3390/vetsci10100611 (PMC10611108; doi:10.3390/vetsci10100611)
Supplement: Supplementary file 1 [file vetsci-10-00611-s001.zip › vetsci-2541648-supplementary.pdf]

Table S1: Details of preoperative findings, complications, survival time, and post-mortem lesions on fishes undergoing cataract surgery. Fish classified as having died to an ocular cause (3/11) were all humanely euthanized due to a loss of vision, anorexia, and a low body condition score, with no other abnormalities identified during post-mortem examination. The general signs observed, such as anorexia, darkening of the body coloration, scoliosis, or wounds, were attributed to one eye to emphasize the chronology of the lesions. This sign was allocated to the last operated eye; however, it is not possible to definitively ascertain that this clinical manifestation is directly related to the designated eye.

Lateralization of the lesions: L, left; R, right.

Systemic clinical signs, listed in italics: A, anorexia; AW, abrasion wound; DIN, dark in color; DAFO, decreased avoidance for obstacles; LBS, low body score; Sc, scoliosis.

Ophthalmic clinical signs, listed in bold: B, buphthalmos; CE, corneal edema; CO, corneal opacity; En, enophthalmos; GB, gas bubble; H, hyphema; M, miosis; My, mydriasis; OD, iris oval distortion; RD, retinal detachment; S, synechia; U, uveitis.

N/A, non-applicable; NS, not specified in records.

	Preoperative evaluation	Complications noted during follow-up				Concomitant problems and survival time			Necropsy	
Fish number (Eye affected)	**At D0<br>(Before surgery)<br>All eyes had mature cataracts.**	**First follow-up<br>[D3-D6]**	**Second follow-up<br>[D7-D14]**	**Third and subsequent follow-ups<br>[D15-D365]**	**Follow-up after a year<br>> D365**	**Concomitant medical problem**	**Live(L)/ Dead(D)**	**Minimal survival time (days)**	**Eye histological lesions**	**Other *post-mortem* lesions**
**1<br>(R)**	**S**<br>*A, DAFO*	**CE, S, U**<br>*DIN*	**CE, S**<br>*DIN, DAFO*	**CE, S**<br>*DIN*	**S**	None	L	1425	N/A	
**2<br>(R and L, surgery the same day)**	*A, DIN, DAFO*	**CE, H**<br>*DAFO*	NS	NA	NA	Testicular tumor (gonadectomy followed by coelomic effusion)	D	27	NS	Coelomitis and septicemia
	A, DIN, DAFO	**CE**<br>*DAFO*	NS	NA	NA					
**3<br>(L)**	*A, DIN*	**S, U**	**S, U, CO**	**S, U, CO, RD**	NA	Nephropathy	D	315	S, RD	Pale posterior kidney
**4<br>(L, then R)**	NS	NS	NS	NS	**RD**<br>*DIN, DAFO*	None	L	1394	N/A	
	A, DIN, DAFO	**CE, GB**<br>*DAFO*	**CE**<br>*DAFO*	NS	NS					
**5<br>(R)**	*A, AW*	**CE, H, GB, U**<br>*A*	**CE, H**<br>*A, AW*	**CO, S, M, En**<br>*Sc*	NS	Testicular tumor	D	440	NS	Distended swim bladder
**6<br>(R, then L)**	*A, AW, DAFO*	NS	NS	NS	M, En, S, retro-orbital mass<br>Sc, AW	Coelomic effusion	D<br>(ocular cause)	1439	NS	AW, distended gall bladder, small kidneys
	**Lens luxation**<br>*A, Sc, AW*	**S, CE, U, OD, H, My**	**S, CE, H**	**CO, H**	**CO**<br>*DAFO, DIN, AW, LBS*					
**7 (R)**	*A, DIN*	NS	**U, GB, H**	**GB, CO**	N/A	None	D	357	NS	Kidney mass
**8<br>(L, then R)**	*A, AW*	**CE**<br>*A*	**CE, B, U**<br>*A*	**CO, B, RD**	**RD, CO**<br>*AW*	None	D<br>(ocular cause)	812	RD	Uroliths, hepatic lipidosis
	A, AW	*A*	**CE**<br>*A*	**CO**<br>*DAFO, LBS, A*	N/A					
**9<br>(R)**	*A, LBS*	**GB**	NS	**CO, S**<br>*DAFO, LBS, A*	N/A	Urolithiasis, cystotomy	D<br>(ocular cause)	133	NS	NS
**10<br>(R)**	**Lens luxation**<br>*A, AW*	**CE, H**	**CE, U, M**	**CE, M, U**<br>*AW*	N/A	None	D	66	Endophthalmitis, RD	AW, abundant cerebrospinal fluid
**11 (R)**	**B**<br>*A, LBS*	**B, CE**	**CE, B**	NS	NS	None	L	1334	N/A	
